# International Age and Use Criteria for Transcatheter and Surgical Aortic Valve Replacement

**DOI:** 10.31083/RCM49368

**Published:** 2026-06-29

**Authors:** Rohit Sharma, Sudarshan Srivats, Fateen Ata, Jeffrey Shuhaiber

**Affiliations:** ^1^Department of Medicine, Mass General Brigham-Salem Hospital, Salem, MA 01970, USA; ^2^Department of Hospital Medicine, Catholic Medical Center, Manchester, NH 03102, USA; ^3^Department of Medicine, Hamad Medical Corporation, 3050 Doha, Qatar; ^4^The Wellman Institute of Photomedicine, Massachusetts General Hospital, Harvard Medical School, Boston, MA 02114, USA

**Keywords:** TAVR, SAVR, age-criteria, comparison, international

## Abstract

Transcatheter aortic valve replacement (TAVR) and surgical aortic valve replacement (SAVR) have transformed the management of severe aortic stenosis across a wide range of patient risk profiles. As populations age and indications extend to lower-risk and younger patients, determining the appropriate role of age in selecting TAVR versus SAVR has become increasingly complex. Current guidelines emphasize individualized decision-making, yet age-based referral patterns remain common in clinical practice. This review examines how age thresholds are applied in contemporary guidance from the United States (US), Europe, and the Asia-Pacific region. Additionally, this review evaluates whether chronological age alone is a defensible basis for referral or treatment selection. This evidence-based narrative review queried PubMed, Embase, and Cochrane Central Register of Controlled Trials (CENTRAL) from January 1, 2013, through December 31, 2025, using the search terms: ("aortic stenosis" AND ("TAVR" OR "transcatheter aortic valve implantation (TAVI)" OR "SAVR")) AND ("age" OR "appropriateness"). English-language abstracts and full texts were screened in duplicate. Eligible studies included randomized controlled trials, national or continental registries, health economic simulations, and clinical practice guidelines that reported age-stratified outcomes or recommendations. Single-case reports, editorials, and animal studies were excluded. Of the 1628 titles screened, 87 full texts were reviewed, and 45 studies were retained. Across regions, guidelines converge on a core principle: age is informative but insufficient in isolation. U.S. guidance generally favors SAVR in patients younger than 65 years or those with a life expectancy greater than 20 years, and favors transfemoral TAVR in patients older than 80 years or with a life expectancy shorter than 10 years. European guidance typically favors SAVR in patients younger than 75 years and TAVR in those older than 75 years. Meanwhile, Asia-Pacific recommendations adopt a similarly individualized approach but place greater emphasis on bicuspid anatomy, rheumatic disease, local health system infrastructure, and cost. Recent data support a cautious approach in younger patients. In observational U.S. analyses of patients younger than 65 years, TAVR use increased substantially despite guideline preference for surgery, and TAVR was associated with higher long-term mortality or higher pacemaker and readmission burdens in selected cohorts. Contemporary randomized data suggest broadly similar outcomes between TAVR and SAVR in older or intermediate-age populations; however, uncertainty persists in younger low-risk patients, particularly those with bicuspid anatomy and long projected survival. Age-based cutoffs should be interpreted as decision anchors rather than rigid rules. The most defensible framework integrates age with life expectancy, valve durability, anatomy, frailty, comorbidity burden, coronary artery disease, feasibility of future valve-in-valve therapy, and patient preferences within a multidisciplinary heart team. Expansion of TAVR into younger populations should remain measured until more robust long-term durability and lifetime management data become available.

## 1. Introduction

Transcatheter aortic valve replacement has moved from a therapy reserved for inoperable or very high-risk patients to a mainstream treatment option across a wide range of surgical risk profiles. That success has created a new problem: procedural eligibility is expanding faster than the long-term evidence base, especially in younger patients with longer life expectancy. As the global population ages and valvular heart disease becomes more prevalent, optimizing patient selection for transcatheter aortic valve replacement (TAVR) and surgical aortic valve replacement (SAVR) is of paramount importance. Although both procedures are well established, expanding indications—particularly into younger and lower-risk populations—have created uncertainty regarding optimal patient selection, especially with respect to age. One critical aspect of this decision-making process is determining the appropriate age cut-off for each procedure. The central question is no longer whether TAVR works. It is which patients benefit most from TAVR, which still benefit more from SAVR, and how age should be weighed against anatomy, durability, and lifetime management [1,2,3].

In this review, we synthesize contemporary guideline recommendations from the American Heart Association/American College of Cardiology, major Asian cardiovascular societies, and the European Society of Cardiology to address two central questions (Table 1). First, we examine how international guidelines differ in their use of age cut-offs and appropriateness criteria when recommending transcatheter versus surgical aortic valve replacement. Second, we evaluate whether the widely applied clinical practice of directing patients younger than 75 years toward surgical referral and those aged 75 years or older toward multidisciplinary heart-team evaluation is supported by current trial evidence, registry data, and durability considerations. The aim is to compare age-related recommendations across the United States, Europe, and Asia-Pacific; integrate newer evidence published in 2024 and 2025; and clarify where age-based thresholds are useful, where they become reductive, and how they should be embedded within a more defensible decision framework.

**Table 1.** **Comparison of guidelines between US, Europe and Asia**.

Criteria	U.S. (ACC/AHA 2020)	Europe (ESC/EACTS 2021)	Asia-Pacific (APSC 2021)
Age cut-off for SAVR	SAVR preferred if <65 years	SAVR preferred if <75 years	SAVR preferred if <75 years, especially if life expectancy >10 years
TAVR preferred	If ≥80 years or life expectancy <10 years	If ≥75 years, or high surgical risk (EuroSCORE II >8%)	If ≥75 years or surgical risk is intermediate to high, and anatomy is suitable
Intermediate-age group decision (e.g., 65–80)	Shared decision making with Heart Team; consider comorbidities, anatomy, preferences	Shared decision making with patient and team, based on surgical risk and anatomy	Shared decision making essential; emphasizes importance of local expertise and resources
Use of risk scores (STS/EuroSCORE)	Considered, but less emphasized than age and comorbidities	Strong reliance on risk scores (STS-PROM, EuroSCORE II)	Supports risk scores but stresses Heart Team judgment due to variability in risk models across Asian populations
TAVR in bicuspid aortic valve	Cautious; not routine	Cautious; generally prefer SAVR	TAVR may be considered with favorable anatomy and expertise, especially in elderly
Procedural access	Transfemoral preferred for TAVR	Transfemoral preferred	Transfemoral preferred; non-transfemoral reserved for experienced centers

ACC, American College of Cardiology; AHA, American Heart Association; ESC, European Society of Cardiology; EACTS, European Association of Cardio-thoracic Surgery; APSC, Asia Pacific Society of Cardiology; STS-PROM, Society of Thoracic Surgeons Predicted Risk of Mortality; TAVR, transcatheter aortic valve replacement; SAVR, surgical aortic valve replacement.

## 2. Methods

Search strategy and selection—Because this review was prepared as an evidence-based narrative (not a formal systematic review), we now specify our process. PubMed, Embase and Cochrane Central Register of Controlled Trials (CENTRAL) were queried (Jan 1 2013 → Dec 31 2025) with (["aortic stenosis" AND ("TAVR" OR "transcatheter aortic valve implantation (TAVI)" OR "SAVR")] AND ["age" OR "appropriateness"]). Abstracts and full-texts in English were screened in duplicate. Inclusion: original randomized controlled trials (RCTs), national/continental registries, health-economic simulations and guidelines that reported age-stratified outcomes or recommendations. Exclusion: single-case studies, editorials and animal work. Priority was given to sources that directly addressed age-stratified outcomes, valve durability, lifetime management, heart-team decision-making, and regional implementation. In total 1628 titles → 87 full texts → 50 studies were retained (See Table 2).

**Table 2.** **The search strategy summary**.

Items	Specification
Date of search (specified to date, month and year)	December 31, 2025.
Databases and other sources searched	PubMed, Embase, and Cochrane Central Register of Controlled Trials (CENTRAL).
Search terms used (including MeSH and free text search terms and filters)	(“aortic stenosis” AND (“TAVR” OR “TAVI” OR “SAVR”)) AND (“age” OR “appropriateness”). Both MeSH terms and free-text keywords were used.
Timeframe	January 1, 2013 to December 31, 2025.
Inclusion and exclusion criteria (study type, language restrictions, etc.)	Inclusion: Original randomized controlled trials, national or continental registries, health-economic simulation studies, and clinical practice guidelines reporting age-stratified outcomes or recommendations. Exclusion: Single-case studies, editorials, and animal studies. Only English-language publications were included.
Selection process (who conducted the selection, whether it was conducted independently, how consensus was obtained, etc.)	Abstracts and full-text articles were screened in duplicate by reviewers. Discrepancies were resolved through discussion and consensus.
Any additional considerations, if applicable	This review was conducted as an evidence-based narrative review rather than a formal systematic review. In total, 1628 titles were screened, 87 full-text articles were assessed, and 45 studies were retained for inclusion.

TAVR, transcatheter aortic valve replacement; TAVI, transcatheter aortic valve implantation; SAVR, surgical aortic valve replacement.

## 3. Section 1: TAVR and SAVR Guidelines in the United States

The United States has been at the forefront of TAVR and SAVR adoption, witnessing significant advancements in procedural techniques and technology. The American Heart Association (AHA) and American College of Cardiology (ACC) have played a central role in issuing guidelines for valvular heart disease, with a focus on appropriate patient selection for TAVR and SAVR.

The current AHA/ACC guidelines recommend TAVR as a Class I indication for patients with severe symptomatic aortic stenosis who are at high surgical risk (Society of Thoracic Surgeons [STS] score ≥8%), age >80 years, life expectancy <10 years (calculated from the 2020 U.S. actuarial life table stratified by sex) [4] or are deemed inoperable [5,6]. Similarly, SAVR is recommended as a Class I indication for patients with symptomatic severe aortic stenosis with low surgical risk (STS score <3%), age <65, life expectancy >20 years [5,7,8].

Regarding age cut-offs, the guidelines do not prescribe a strict numerical threshold for TAVR or SAVR candidacy. Instead, they emphasize individualized patient assessment based on factors such as frailty, life expectancy, and overall health status. This patient-centered approach recognizes that chronological age alone does not dictate treatment appropriateness.

Recent studies comparing TAVR and SAVR outcomes in the United States have shown favorable results for both procedures, with TAVR demonstrating comparable or superior outcomes in certain patient subsets. The evolution of TAVR technology has expanded its use to younger and lower-risk patients, blurring the lines of age-based selection criteria. It is prudent to note that durability data for SAVR extends more than 50 years [9]. Data for TAVR durability is currently available for up to 10–12 years, primarily from the NOTION trial and PARTNER 3 5-year follow-up [10,11,12].

2020 ACC/AHA guidelines have recommended (Class of recommendation - COR1) for a shared decision making approach when considering intervention for TAVR or SAVR [1]. Despite this, some physicians remain proponents of the age-based referral approach. Age as a surrogate of life expectancy has been used based on the US actuarial life table [4]. They argue that patients below 75 years are generally more robust and may tolerate the invasiveness of SAVR better, warranting an initial referral to surgeons. Conversely, patients above 75 years might have higher comorbidities and benefit from the collaborative decision-making facilitated by heart teams. 2020 AHA/ACC guidelines suggests SAVR (COR1) for age <65 and TAVR (COR1) or SAVR (COR2a) for age >80. Earlier trials have shown equivocal outcomes between TAVR and SAVR with average patients age in min 80s [13,14]. Patient in late 70s to mid 80s with intermediate surgical risk also showed no difference mortality or disabling stroke TAVR vs. SAVR [15,16,17]. More recent RCT included patients in mid 70s showed non-significant difference in mortality between the 2 but long term follow up was not available [18] (Table 3, Ref. [11,14,19,20,21,22,23]).

**Table 3.** **Age cut-off used in recent RCTs with outcomes**.

Study name and authors	Age, inclusion criteria	Mean/median age	Outcomes
UK TAVI trial investigators, Toff et al. [19]	70 years or older with severe symptomatic AS, moderately increased peri-operative risk	Median age 81 years [IQR, 78 to 84 years]	TAVR noninferior to SAVR for 1 year all-cause mortality
Evolut low risk trial investigators, Forrest et al. [20]	Low risk patients with severe aortic stenosis	Mean age 74 years	At 3 years, durable benefits for TAVR vs. SAVR in terms of all cause-mortality or disabling stroke
SURTAVI trial investigator, Van Mieghem et al. [21]	Intermediate risk severe aortic stenosis	Mean age 79.8 years	At 5 years, clinical outcomes similar in terms of mortality or disabling stroke
NOTION trial, Thyregod et al. [11]	Low risk patient with severe aortic stenosis	Mean age 79.1 years	At 5 years, all-cause mortality, stroke or MI was not different
PARTNER 3 trial, Mack et al. [22]	Low risk patient with severe aortic stenosis	Mean age 73 years	At 1 year, rate of composite death, stroke, rehospitalization lower with TAVR
STACCATO trial, Nielsen et al. [23]	Low risk patient with severe aortic stenosis, age >75 years	Mean age 81 years	Prematurely terminated due to excess events in TAVR group at day 30
CoreValve U.S. pivotal high risk, Gleason et al. [14]	High risk patients with severe aortic stenosis	Mean age 83 years	Mid-term survival and stroke similar between TAVR and SAVR

RCTs, randomized controlled trials; AS, aortic stenosis; MI, myocardial infarction.

The guidelines are ambiguous for age 65–80 with either SAVR (COR1) or TAVR (COR1) [1]. RCTs show TAVR’s efficacy in high-velocity severe aortic stenosis (AS) (Stage D1), while observational data suggest its promise in low-flow, low-gradient severe AS (Stages D2, D3). TAVR offers lower mortality, shorter hospital stay, quicker recovery, less atrial fibrillation (AF), bleeding, and pain than SAVR. SAVR, however, has lower risks of paravalvular leak, valve reintervention, and pacemaker need. Decision between SAVR or TAVR for 65–80-year-olds depends on factors like vascular access, comorbidities, expected post-aortic valve replacement (AVR) functionality and survival, and patient preferences. Choosing between mechanical/bioprosthetic SAVR and TAVR involves considering valve durability, as transcatheter valves’ longevity beyond 5–6 years is uncertain [7]. Factors favoring SAVR include younger age or longer life expectancy, valve anatomy (bicuspid aortic valve (BAV), subaortic calcification, rheumatic heart disease (RHD), small or large aortic annulus), cardiac co-morbidities (coronary artery disease (CAD) requiring coronary artery bypass grafting (CABG), Severe primary mitral regurgitation (MR), aortic dilation, AF, Septal hypertrophy requiring myomectomy) [1]. Factors favoring TAVR include older age/shorter life expectancy, calcific AS with trileaflet valve, severe calcification of ascending aorta and other co-morbidities (severe lung, renal or liver disease). Other important factors for consideration include frailty, procedure specific impediments and patient’s preference.

Meta analysis of 7 landmark trials for severe symptomatic aortic stenosis shows decrease in stroke and all-cause mortality for patients who underwent TAVR across age spectrum [24]. It is worth noting that follow up duration for this meta-analysis was only 2 years, which is a significant limitation when it is known that SAVR performance is tested in terms of long term mortality. Retrospective data from Medicare beneficiaries from 2012–2019 shows an overall trend of migration of patients from SAVR to TAVR [25]. It is noted that TAVR has been expanding to lower risk population but this has not affected SAVR outcomes in terms of short and long term mortality [25]. It was also noted from a study in Canada that utilization of heart team, which is recommended by 2020 AHA/ACC (COR1); has been on a decline from 69.9% to 41.1% [26]. The approach of not utilizing a multidisciplinary team in North America is highly concerning.

Newer U.S. data make the tension clearer. In a multicenter observational analysis of patients younger than 65 years, TAVR use rose from 7.1% in 2013 to 54.7% in 2021, despite guideline preference for surgery in most patients within that age range [27]. After matching, early mortality was similar, but TAVR was associated with higher 6-year mortality and more new pacemaker implantation than biologic SAVR [27]. A complementary analysis of balloon-expandable TAVR in the low-risk era showed that patients younger than 65 years represented a small but clinically distinct subgroup with greater comorbidity burden and worse one-year outcomes than older TAVR recipients, suggesting that younger age in real-world practice often marks clinical complexity rather than straightforward lower risk [28]. As Table 2 shows, the guideline itself supports individualized treatment, not age-only triage.

## 4. Section 2: TAVR and SAVR Guidelines in Asia

Age-based recommendations in Asia have historically been harder to generalize because the region is heterogeneous in valve morphology, rheumatic disease burden, institutional capacity, reimbursement, and operator experience. The 2024 Asian Pacific Society of Cardiology position statement is therefore an important update [3]. It recognizes the rapid uptake of TAVI across the region, but it also emphasizes bicuspid valve disease, resource disparities, and country-specific life expectancy as major determinants of appropriateness [3]. Leading cardiovascular societies in Asia, such as the Japanese Circulation Society (JCS) and the Chinese Society of Cardiology (CSC) play a crucial role in issuing guidelines for TAVR and SAVR. Indian Heart Association has not issued guidelines for Indian subpopulation yet but tends to follow the AHA/ACC guidelines.

Similar to the US guidelines, Asian guidelines recommend TAVR for patients with severe symptomatic aortic stenosis at high surgical risk or considered inoperable. However, differences in age cut-offs and appropriate use criteria exist among Asian regions. Some guidelines adopt a more conservative approach, favoring SAVR over TAVR in patients below a certain age, while others endorse individualized assessment for each patient.

In Japan, Studies have demonstrated comparable 30 day mortality between SAVR and TAVR (1.9% & 1.7%, respectively) [29,30]. JCS recommends SAVR should be considered for younger patients and TAVR for older patients [31]. While JCS does not give a strict age cut off, guidelines have mentioned prioritization of TAVR for age >80 and SAVR for age <75 [31]. Multicentric retrospective data from TAVR registry in China showed strikingly low mean age of patients 73.8 ± 6.5 years and 1 year cumulative mortality of 4.5%, which is higher than other reported studies [32].

Adoption of TAVR in India seems to be significantly behind as compared to USA, Europe, Japan and China. In a 2020 report it was noted that only 30 centers perform TAVR in India [33]. But as of late 2024, over 200 centers perform TAVR in India, indicating significant growth since 2020. Limited retrospective data is available from India. Wide variety of reasons could lead to high one year mortality such as operator proficiency, anatomical factor such as high prevalence of BAV, smaller body surface area, small diameter of common femoral and iliac artery [33]. There are multiple unique challenges in India making TAVR harder to implement widely. Cost is estimated to be 35,000 USD in 2020 which is extremely high from the South East Asian economic perspective [33]. Apart from this amongst Asian population it is worth noting that coronary ostia take-off is low and annulus may be smaller which poses challenge of Asia pacific population in general [34].

Cost of TAVR remains high in Asian subcontinent as compared to SAVR [35]. A simulation study from China suggested that an approximate 65% reduction would be required for TAVR to be cost-effective [36]. Similar trends have been voiced by experts in India although there is lack of clear health care cost utilization data. It has also been noted that although TAVR cost is higher as compared to SAVR in the US, there is a trend for decreased cost for TAVR overtime [37]. This trend could potentially follow in Asia as well with cheaper TAVR valves in future.

Age-based referral decisions in Asia must also account for cultural factors and patient preferences. In some Asian cultures, a strong emphasis on family and societal values may influence treatment choices. Additionally, healthcare systems with varying levels of infrastructure and expertise in TAVR may limit its availability, impacting referral patterns.

## 5. Section 3: TAVR and SAVR Guidelines in Europe

Europe boasts a diverse landscape of healthcare systems and demographic profiles, reflecting substantial variations in the management of valvular heart disease. The European Society of Cardiology (ESC) has been instrumental in formulating guidelines to address the complexities of TAVR and SAVR in European countries.

ESC guidelines recommend TAVR as a Class I indication for patients with severe symptomatic aortic stenosis at high surgical risk or considered inoperable [2]. Similarly, SAVR is recommended as a Class I indication for patients with symptomatic severe aortic stenosis and low surgical risk [2]. 2021 ESC/European Association for Cardio-Thoracic Surgery (EACTS) also recommends a heart team evaluation for the choice of intervention. The guidelines go a bit further with age and recommend SAVR for age <75 at low surgical risk or those patients unsuitable for TAVR but operable [2]. TAVR is recommended for patients >75 years or those who are unsuitable for SAVR or high surgical risk. The age-based recommendation is based on expert consensus which the 2021 ESC/EACTS guidelines recognize. According to expert consensus, age as a surrogate marker for average life expectancy did not affect 1–2 years mortality outcomes in low risk RCTs [2].

Special emphasis for age is placed in 2021 ESC/EACTS with key consideration being durability of prosthetic heart valve. We note that long term durability (up to 8 years) of devices used for TAVR were largely restricted to older population who were high or intermediate risk [10,38,39,40]. This is contrast to the fact that surgical bioprosthetic valves durability is well established beyond 10 years [9]. 2021 ESC/EACTS acknowledges ambiguity over durability of valves used in TAVR. In PARTNERS 3 trial, TAVR group using Balloon expandable valve had higher re-intervention as compared to SAVR at 5 years [15]. When using SAPIEN 3 device, rate of structural valve deterioration was found to be similar [41].

Retrospective data from Europe seems to be concerning. For instance, from SwissTAVI registry should higher standardized mortality ratio for patients who underwent TAVR age <70 [42]. Another retrospective study from a single center in Oslo should provide satisfactory 5-year survival outcomes for all SAVR patients [43]. The 5-year mortality distribution was noted to be 10%, 20% and 34% in patients aged <70 years, 70–79 years and ≥80 years, respectively [43]. Europe has been at the forefront of TAVR research and development, with numerous clinical trials and real-world studies contributing to the evidence base. Age cut-offs and appropriate use criteria in European guidelines are generally aligned with those of the AHA/ACC guidelines, emphasizing individualized decision-making. However, variations in healthcare systems and cultural factors among European countries can lead to differences in the implementation of these guidelines.

New evidence from the NOTION-2 program is especially important because it moves closer to the population clinicians worry about. In a substudy of low-risk patients aged 70 years or younger, outcomes were similar between TAVR and SAVR in tricuspid aortic stenosis but worse with TAVR in bicuspid disease, reinforcing the point that anatomy can outweigh age alone [44]. Three-year follow-up of NOTION-2 also showed no clear advantage for routine TAVR over surgery in younger low-risk patients, with a signal of higher event rates in bicuspid stenosis [45].

In some regions, the concept of heart teams, comprising interventional cardiologists, cardiac surgeons, and other specialists, has been embraced to facilitate collaborative decision-making for complex cases. The heart team approach allows for comprehensive assessment of patient factors beyond age, leading to tailored treatment recommendations.

## 6. Section 4: Physicians’ Perspectives on Age Cut-Off and Referral Decisions

The debate over age cut-off and referral decisions for TAVR and SAVR is complex and multifaceted, involving various stakeholders, including physicians, patients, and healthcare administrators. Some physicians argue that referring patients below the age of 75 years to surgeons first may allow for better long-term durability of SAVR, given the potential for younger patients to outlive the durability of bioprosthetic valves used in TAVR. Additionally, younger patients may be more suitable candidates for complex surgical procedures, potentially yielding superior outcomes with SAVR.

On the other hand, advocating for heart teams to manage patients above the age of 75 years is grounded in the belief that a multidisciplinary approach can consider the intricacies of advanced age, comorbidities, and frailty, ensuring patient-centered care. The heart team model fosters shared decision-making, promoting a balanced evaluation of benefits and risks for both TAVR and SAVR, ultimately leading to treatment decisions aligned with patients’ preferences and values.

However, the age-based referral approach is not without criticisms. Critics argue that it risks oversimplifying the decision-making process, potentially leading to suboptimal patient outcomes. Age alone cannot account for the heterogeneity of patient profiles, and many elderly patients can successfully undergo TAVR with excellent results. Relying solely on age as a criterion may overlook the importance of frailty, cognitive status, and patient preferences in treatment selection.

## 7. Section 5: Ethical Considerations

Ethical challenges in the application of clinical guidelines for elderly patients are compounded by significant barriers to effective communication and shared decision-making, especially in diverse regional healthcare contexts. Cognitive decline, hearing impairment, and limited health literacy frequently hinder older adults from fully understanding complex medical procedures. In many cultures where medical hierarchies are deeply ingrained, elderly individuals often defer entirely to physician authority, reducing patient autonomy. This issue is further exacerbated in low-resource settings, where high patient volumes and time constraints limit the ability of clinicians to engage in detailed discussions. Physicians may experience frustration when explaining intricate interventions to cognitively impaired patients under pressure, increasing the risk that patients may consent without comprehending the full scope of risks and alternatives. To mitigate these concerns, it is ethically imperative to adopt communication strategies that support informed consent—such as simplifying medical language, utilizing visual aids, and involving family members or caregivers in the decision-making process. These measures are essential to ensure that care remains patient-centered, respectful, and ethically sound across all healthcare settings.

## 8. Section 6: Complexities of the TAVR vs. SAVR Debate

The TAVR vs. SAVR debate is multifaceted, extending beyond age cut-offs and appropriate use criteria. Patient risk stratification must consider multiple factors, including anatomical considerations, comorbidities, frailty, cognitive status, and patient preferences. TAVR has demonstrated favorable outcomes in elderly and high-risk patients, while SAVR remains the gold standard for younger, lower-risk patients. Main risk with TAVR revolves around re-intervention if procedure happens in younger population, whereas increasing use of bioprosthetic valve also increases risk of re-intervention in the long run. Multiple complications are attributed to TAVR which are either rare or less frequent in SAVR such as paravalvular leak, need for pacemaker, conduction defect and difficult coronary artery access [46].

The main limitation of bioprostheses is their durability, with structural and nonstructural deterioration, thrombosis, and endocarditis as key failure modes [46]. European guidelines recommend mechanical prostheses for patients under 60, while biological ones are suggested for those over 65, mirroring American guidelines that set the age preferences at below 50 and over 65, respectively. However, the evidence level for these recommendations is low, highlighting the importance of individual patient factors. Long-term data on TAVR durability, particularly for younger patients, is limited, although recent findings show no significant differences in outcomes between TAVR and SAVR in low-risk patients over an 8-year follow-up. One Discreet event Simulation study indicates that TAVR valve durability needs to be significantly compromised—specifically, 70% less than that of SAVR valves—to impact life expectancy negatively in older low-risk patients, supporting the current evidence favoring TAVR’s durability for this demographic [47]. However, for younger patients, the threshold for acceptable TAVR valve durability is lower, underscoring the necessity of considering valve longevity alongside other clinical factors in these individuals. This suggests that while TAVR remains a viable option for older patients based on durability, the choice of TAVR vs. SAVR in younger patients requires careful deliberation of expected valve life span against the backdrop of patient-specific life expectancy and clinical profile. A meta-analysis comparing Valve-in-Valve (ViV) TAVR to redo SAVR revealed that ViV TAVR offers several advantages including lower rates of 30-day mortality, major bleeding, and shorter hospital stays, without significant differences in 1-year mortality compared to redo SAVR [48]. However, ViV TAVR has higher rates of severe patient-prosthesis mismatch (PPM) compared to redo SAVR, which is linked to higher mortality and heart failure hospitalizations in some studies, though this was not reflected in immediate post-procedural mortality in the current data. This analysis underscores ViV TAVR as a viable, less invasive alternative for frail and elderly patients requiring valve reintervention, while recognizing that certain anatomical and procedural factors may still favor redo SAVR in specific cases.

The future of TAVR and SAVR involves a nuanced interplay of various factors across Europe, Asia, and America.

TAVR is poised for continued growth due to its minimally invasive nature and shorter recovery times, potentially surpassing SAVR in utilization [49]. Advancements in TAVR technology, such as improved valve design and delivery systems, alongside expanded eligibility criteria, may accelerate this trend. Clinical trials demonstrating TAVR’s efficacy in intermediate and low-risk patients indicate its potential for broader adoption [18].

SAVR, while facing competition from TAVR, remains indispensable for certain patient populations and complex cases and in resource-poor countries such as Africa or India where the cost of SAVR is more appealing. Refinement of surgical techniques and post-operative care aims to optimize outcomes and maintain SAVR’s relevance alongside TAVR. It is worth noting that Outcomes from early TAVR trials (e.g., PARTNER 1) may not reflect improved safety of current-generation devices such as SAPIEN 3 or Evolut PRO [41].

Geographical disparities in TAVR and SAVR adoption are anticipated, driven by healthcare infrastructure, reimbursement policies, and cultural preferences. Regions with aging populations (such in the WEST) and advanced healthcare systems may favor TAVR, while others may rely more on SAVR due to resource constraints or differing healthcare priorities [50].

Research, clinical trials, and long-term studies will continue to inform the landscape of aortic valve replacement therapies, influencing clinical practice guidelines and reimbursement policies. Cost considerations and healthcare economics will play a pivotal role in shaping the balance between TAVR and SAVR utilization. Shared decision making must avoid unwarranted influence. Recent North-American surveys show that 41% of patients <65 y cited ‘doctor recommendation’ as the sole reason for choosing TAVR, highlighting the need for a structured, standardized consent script that discloses pacemaker risk, coronary-access limitations and uncertain durability. In patients <65, TAVR is increasingly pursued based on patient preference leading to poor outcomes. TAVR in <65 low-risk patients presents challenges including increased risk of pacemaker implantation, coronary access issues, and durability uncertainties. TAVR-in-TAVR procedures may pose anatomical constraints [27,51,52]. However, this can be influenced by operator bias. Standardized informed consent and structured decision aids should be developed to mitigate this risk. To improve quality-of-life evaluation, patient-reported outcome measures (PROMs) should be routinely incorporated in trial design and national registries. Redo SAVR and ViV TAVR strategies should be pre-planned in younger patients. Anatomical considerations such as bicuspid valves, annulus size, and coronary height influence feasibility. Based on guideline comparisons, registry data, and emerging patient trends, we propose a personalized approach that balances procedural risks, life expectancy, anatomical feasibility, and patient values (Table 4).

**Table 4.** **Decision framework for patients aged 65–80 years**.

Factor	Favors SAVR	Favors TAVR	Neutral/either
Annulus/root anatomy	Bicuspid valve, heavy LVOT calcium, small annulus requiring root enlargement	Trileaflet, adequate annulus, porcelain aorta	Mild Mitral Annular Calcification
Concomitant disease	CAD requiring CABG, severe MR needing repair	Severe COPD, liver cirrhosis, prior chest radiation	Mild CAD amenable to PCI
Frailty/recovery goals	Robust, willing to undergo sternotomy	Prefers quicker recovery, limited social support for rehab	—
Anticipated valve-in-valve options	Age <70 years, life expectancy >20 years	Age >75 years, ViV TAVR feasible in future	70–75 years (shared decision)

LVOT, left ventricular outflow tract; CAD, coronary artery disease; CABG, coronary artery bypass grafting; MR, mitral regurgitation; COPD, chronic obstructive pulmonary disease; PCI, percutaneous coronary intervention; ViV, Valve-in-Valve.

Overall, the future directions of TAVR and SAVR involve a complex interplay of technological advancements, clinical evidence, healthcare policies, and patient preferences, driving ongoing evolution in the management of aortic valve disease worldwide. Table 1 shows some of the factors that could aid in clinical decision.

## 9. Conclusion

The age cut-off and appropriate use criteria for TAVR and SAVR in the US, Asia, and Europe are evolving concepts that must be approached with careful consideration. The latest guidelines from the AHA, ACC, Asian cardiovascular societies, and the ESC emphasize the importance of individualized patient assessment. While some physicians advocate for an age-based referral approach, the complex nature of the TAVR vs. SAVR debate necessitates shared decision-making and a multidisciplinary heart team approach.

Patient outcomes should be at the core of treatment selection, and a nuanced evaluation of age, comorbidities, anatomical factors, and patient preferences is essential. Continued research, collaborative efforts, and evidence-based practices will shape the future of aortic valve disease management, optimizing patient outcomes and ensuring the best possible care for all individuals affected by valvular heart disease. Continued global data reporting, especially from Asia, is essential to update these criteria.

These limitations highlight the need for long-term, age-stratified durability studies, prospective evaluation of lifetime valve management strategies, and broader global registry participation, especially in underrepresented regions. Based on cross-regional guideline comparisons, real-world registry data, and emerging patient trends, we propose a personalized approach to valve intervention that balances procedural risk, anticipated valve durability, anatomical considerations, and patient values, while accounting for lifetime valve planning. Continued research, collaborative international efforts, and systematic data reporting—particularly from Asia—will be critical to refining age-appropriate use criteria and informing future guideline updates. Ultimately, moving away from rigid age-based referral toward structured, multidisciplinary decision-making offers the most ethical and evidence-based path forward for clinicians, health systems, and policymakers managing severe aortic valve disease.
